# Facing Shadows: working with young people to coproduce a short film about depression

**DOI:** 10.1186/s40900-018-0126-y

**Published:** 2018-11-27

**Authors:** Valerie Dunn, Sally O’Keeffe, Emily Stapley, Nick Midgley

**Affiliations:** 1 Creative Research Collective, Cambridge, UK; 2 Research Department of Clinical, Educational and Health Psychology, University College London, Cambridge, UK; 3 Child Attachment and Psychological Therapies Research Unit (ChAPTRe), Anna Freud National Centre for Children and Families, Cambridge, UK

**Keywords:** Participation, Involvement, Creative, Young people, Animation, Research, Depression, Coproduction

## Abstract

**Background:**

IMPACT (Improving Mood with Psychoanalytic and Cognitive Therapies) is a multi-centre randomised controlled trial of three therapeutic interventions for the treatment of depression in young people. IMPACT- My Experience (IMPACT-ME), a qualitative research study, followed up a sub-sample of families involved in IMPACT to explore young people’s experiences of therapy and depression. Members of the IMPACT-ME steering group, who brought their own experiences of depression and engaging with mental health services, were keen to find ways to provide information about depression and help-seeking beyond traditional academic audiences, specifically to other young people experiencing depression and wondering where to turn: their chosen medium was film. Here we describe and reflect on the four-day coproduction workshops in which researchers, young people and film-makers coproduced ‘Facing Shadows’, a short animation about depression and therapy (https://www.youtube.com/watch?v=LdmRPKUhNEY).

**Main body:**

We outline the process, focusing on the four-day creative, collaborative workshop in which young people shared their experiences, decided on the tone, tenor and message of the film, identified their primary audience and produced the bulk of the audio and visual material. The adults acted as facilitators: developing a creative, collaborative learning environment in which trusting relationships could flourish, as well as offering guidance, instruction, advice and support. To date the film has been viewed around 12,000 times on YouTube. The young people learned new skills, felt listened to and enjoyed the process. They produced a film which sends a hopeful message to other young people, ‘… *that they are not alone’.*

**Conclusion:**

We reflect on the creative participatory workshop approach which transformed the project from dissemination to an insightful learning experience for young people and researchers alike.

## Plain English summary

A group of young people who had been diagnosed with depression were members of a participation group on a clinical trial comparing treatments for depression in young people - the IMPACT Study (Improving Mood with Psychoanalytic and Cognitive Therapies). They identified a need for information about depression and therapy to help other depressed young people who may not know where to turn. They thought that a short film would be an effective medium. Seven young people worked with researchers and film-makers to make Facing Shadows, a short, animated film. Young people attended a four-day creative workshop, where they explored the issues using role plays, drawing, animation, film-making, sound effects and discussions. They recorded conversations and produced artwork for the film. The adults and young people established strong, trusting relationships, young people learned new skills and researchers gained new insights. Young people were keen that their film should send a hopeful message ‘… *to people who are suffering from depression and are really low - that they are not alone’.* The film-makers also made a Behind the Scenes film showing how the films were made. In this paper we describe how the film was made and offer learning points for researchers wishing to carry out similar projects.

## Background

The United Nations Convention on the Rights of the Child [1] enshrines the rights of children and young people to have their views listened to and taken into account. It provides the ethical and legal basis for the active participation of the young in social care, education, health and research. Young people want to be listened to, taken seriously and for their input to make a difference [2]. It is incumbent upon researchers then to devise engaging, relevant ways of working with young people with meaning and purpose, and to avoid tokenism. Involvement which recognises the young as ‘experts in their own lives’ offers researchers new insights and young people the opportunity to learn new skills and knowledge, gain self-confidence, and a sense of empowerment [3, 4].

IMPACT-My Experience (IMPACT-ME, run by NM, SOK, ES) [5], was the qualitative arm of a large, multi-centre randomised controlled trial, IMPACT (Improving Mood with Psychoanalytic and Cognitive Therapies) which tested the efficacy of treatments for depression in young people [6, 7]. IMPACT-ME explored experiences of depression, therapy and recovery with young people and their parents. Initially, Patient Public Involvement (PPI) on the study took the form of young people and parents sitting on the study steering group. This group took an active part in reviewing study information, advising on recruitment, measures and dissemination. As trusting relationships were established, members of this group began to identify a need to provide accessible, relevant information about depression and therapy to young people in the wider population. They identified film as an ideal medium to speak to their peers. The young people were motivated by a desire to inform, ‘*I know so many people going through the same thing and they don’t get help or recognise it*…’; to transform, *‘…change the way that depression and mental health is treated*’; and to help others, *‘if this film helps just one individual, I’ll be delighted*’; and ‘*I hope that the film sends out a message to people who are suffering from depression … that they are not alone’*.

In this commentary we outline the resulting project which culminated in three films: an animation coproduced with young people about the experience of depression and therapy; a Behind the Scenes film which provided the back-story for funders, researchers and others interested in the process; and a short film in which the parents share their experiences of caring for a depressed child, to offer support to other parents in similar positions (not discussed in this commentary). We outline our rationale, the coproduction workshops, and reflect on our learning. In the absence of a full project evaluation, we draw on discussions and interviews recorded during the workshops, for the films and for a MSc thesis (Mellor, 2015, unpublished) as well as field notes and debrief discussions.

## Project development

In thinking how to support this project, IMPACT-ME researchers (NM, SOK, ES) approached the first author (VD), then a mental health researcher at the University of Cambridge, Dept of Psychiatry and co-founder of the Creative Research Collective (CRC), an eclectic group consisting of a researcher, film-makers and a workshop/drama practitioner. The group had co-produced an award-winning trilogy of films with young people in care [8–12] and employed creative, participatory workshop approaches which seemed appropriate to this project. Before any firm commitments to the project were made, an exploratory meeting gave young people the chance to meet – effectively to audition - the CRC team. At this informal meeting, the team described their approach, previous projects and showed films to illustrate their style. Young people asked questions about practicalities, workshop activities and timing. Afterwards the young people opted to work with the CRC team. A ‘taster session’ was arranged where interested young people who had been part of IMPACT-ME could find out more, before deciding whether to participate.

### The taster session

Twelve young people who had been part of the IMPACT-ME study, including the three members of the IMPACT-ME steering group, attended a half-day project taster workshop. The aims were to provide an insight into the animation process, consider the film’s audience and key messages and discuss practicalities such as timing and availability. We showed and discussed a medley of animated film clips to illustrate a range of creative possibilities. Young people experimented with basic animation and sound recording techniques to produce a very simple short taster film. The session served as an ice-breaker and gave us an idea of each young person’s skills and interests. Seven of the 12 young people continued to be involved, along with the three parent members of IMPACT-ME steering group. Interestingly, the taster session also served to enthuse and reassure the researchers: “… *everybody thought it was going to work after the taster day and the excitement of the young people was palpable’; ‘… it’s a success, I feel hopeful’;* and *‘it was great for seeing how it would work with the young people’.*

## The rationale behind the creative workshops

The creative workshops drew on the principles of Participatory Research (PR). The skills and experiences of all relevant stakeholders are assigned equal value, irrespective of age or status. PR is collaborative and democratic, aiming to redress the power imbalances [13]. PR provides time and space for exploration, discussion, reflection and creative thinking. The approaches, which can be engaging for young people, use a range of creative and arts-based techniques, flexible and adaptable in different settings, with different groups. For example, photography, drawing, writing and film can be used to stimulate, or be the end produce of, thought, exploration and discussion. Such techniques are applicable throughout the research process to identify questions, collect data, analyse and interpret, represent and disseminate. For example, film has become a popular medium to make accessible complex concepts, procedures and information [14–17]. The films are often based on information generated in focus groups.

Creative techniques offer opportunities to draw on emotion and imagination [18] and do not rely solely on participants’ verbal or written capabilities, *‘…some knowings cannot be conveyed through language’* [19]. Participatory Theatre, for example, prioritises confidence building, the creation of safe environments and positive team dynamics. Games, role plays, mask work, metaphor, drawing, mapping and drama, offer opportunities to explore complex topics, which may be beyond the reach of words [20, 21]. For example, young people on the BRIGHTLIGHT Study [14], which focuses on specialist services for young people with cancer, collaborated with a youth theatre group. Through a series of workshops, the young actors were helped to ‘experience what it’s like to have cancer as a young person and to understand the services and care they receive…’. The theatre group then used their learning to develop ‘There is a Light: BRIGHTLIGHT’, a piece of improvised theatre which toured the UK.

## The four-day workshop

During the Easter break, seven young people, aged 16–19, three parents, four researchers and five CRC members gathered for a four-day workshop, steered by workshop facilitator, Tom Mellor (TM) who role it was to keep time, adjust the pace, ensure responsivity and sensitivity, scaffold discussion and manage difficulties.

Paramount was to create a safe, trusting, collaborative environment to facilitate sharing and learning, generate ideas, develop skills and achieve the agreed goals. Workshop days were designed to maximise involvement, particularly to include less confident young people. To that end adults and young people worked, played games, lunched and spent breaks together.

Workshop days were loosely timetabled and activities were carefully balanced to maintain momentum, energy and interest. The schedule was flexible to ensure responsiveness to individual needs and emerging topics. A range of creative and arts-based activities, such as role play, mask work, games, drawing and metaphors, were used to facilitate discussion and creative thinking. Working individually, in pairs and as a whole group, young people explored and shared their experiences, ideas and vision for the film and produced the audio and visual material for the screen.

Young people learned basic animation techniques with LH, participated in live-action filming with Andy Dunn (AD) and produced sound effects, recorded interviews and conversations with Mark Simm (MS). Table 1 shows our work schedule for day one of the four days.

**Table 1:** Facing Shadows: workshop plan day 1

09.45	15 m	Arrival, settling in, coffee etc.…
10.00	20 m	Welcome and introductions, housekeeping, confidentiality, why we’re here
10.20	15 m	Ice breaker/warm-up activity
10.35	15 m	Animation: rationale, the possibilities/limitations, techniques, preferences, anonymity, discussion (LH show a range of illustrative examples).
10.50	15 m	Introductory exploration, discussion of the themes.
11.05	75 m	Visualising depression: metaphors, make flip books but also list/store ideas
12.20	40 m	Lunch
13.00	30 m	Intro to the afternoon. Exercise to consider helpful/unhelpful responses to depression – dra*w*/write
13.30	60 m	Round table – therapy: expectations vs reality, concerns, language, environment, therapist, timing etc.
14.30	20 m	Experience of therapy: writing exercise, spread out on floor/ table, assorted paper & pens, write as many/few memories of therapy without pause or block
14.50	10 m	Break
15.00	60 m	In parallel: 1. Individually/pairs draw aspect of therapy environment (clinic, consultation room, waiting room?); 2. In pairs, with AD, film portraits
16.00	15 m	Round up, reflect, review, questions, concerns/problems, day 2 intro
16.15		LH to test acetate, paint with projector

Early on the young people made key decisions about the tenor and message of the film: they wanted it to be realistic about the experience of depression and its impact on every-day life, positive and encouraging about the benefits of seeking and accepting help and clear about the heterogeneity of depression [22]. Six key questions were identified around which the film was structured: *What is depression? How did you know you were depressed? How did it affect your life? Did you speak to anyone? What was therapy like? Do you have any advice for other young people?* The young people drew up a shortlist of possible titles and Facing Shadows won the poll.

Audio for the film was recorded at round-table discussions, candid chats during activities, set-piece paired conversations recorded in a designated quiet room, and sound-effect sessions. All young people provided written consent to be recorded, were always aware when this was happening and were able to withdraw consent for any part of their audio to be used in the film.

### Confidentiality, support and ethics

all young people provided written, informed consented to take part in the project, including consent to be recorded. We asked each young person to complete an ‘about me’ form which provided the opportunity to make us aware of any health or practical needs. At the outset, a confidentiality agreement, which covered social media use and safeguarding, was agreed with all participants.

As a general rule the CRC team recommends that young people are not identifiable on screen. Animation facilitates this, protecting identities of the young people. However, the combination of voice and image, might mean a young person would be identifiable to a viewer familiar with them. We asked the group to give this careful consideration. The photographic and silhouette images were carefully checked to ensure that the young people were happy with the level of anonymity. The group decided to be listed by first names only in the film credits.

The emotional health and wellbeing of the young people was a priority for everyone involved. The topics under discussion may well have acted as triggers for some of the most vulnerable. Indeed, on occasions, individuals did withdraw slightly after speaking about their own depression. Young people were well supported. All the adults were vigilant, sensitive and supportive. A designated quiet space was available for ‘time out’. At the end of each day, researchers and the creative team held debrief sessions to discuss concerns to have arisen during the day.

As the project was not conceived as a research study, approval from a research ethics committee was not sought. However, as experienced practitioners working with vulnerable young people, ethical considerations were given priority. In addition to the above, all adults were DBS checked and experienced at working in public spaces, and so cognizant of health and safety requirements and covered by public liability insurance.

### Making sense of the material

the final editing stage was led by LH who amassed the material in her studio, to select and sequence audio and visual material, finish animating the young people’s drawings and live action sequences and structure a rough cut of the film. LH had been fully immersed in the workshops and the ongoing dialogue with young people through which the tone, content and direction had been established. This informed her decisions and ensured fidelity to the young people’s vision. Aesthetic considerations were important to do justice to the young people’s work and to create a visually arresting and eloquent film for young audiences, as LH explained, ‘*for me, it boils down to making sure that we use all the precious artwork, in conjunction with a selection of the voices and sounds, and from these trying to make a narrative that represents the themes and discussions of the workshops. Editing decisions are about finding a truthful voice and inclusion of all the different points of view*’.

We reconvened to review the rough cut giving young people the opportunity to input, ask questions, suggest changes and withdraw images or audio with which they were.

unhappy. Finally, based on this input, LH produced a final cut of Facing Shadows.

### Post-production

Facing Shadows was premiered at the British Film Institute (BFI) which was hired at a concessionary rate. The event was hosted by mental health campaigner, Jonny Benjamin M.B.E. The young people held a Question and Answer session to a large, invited audience. We were fortunate to be able to capitalise on the high social media profile of the Anna Freud National Centre for Children and Families. Through our active social media campaign, the actor Stephen Fry, who has actively campaigned to promote awareness of depression, tweeted his support for the films. This resulted in thousands of views within hours. Two young people made television appearances to talk about the project and several local newspapers also covered the story. The film has featured on a number of key mental health websites, including Sane and YoungMinds. At the time of writing, Facing Shadows has around 12,000 YouTube views.

## Reflections and learning

The National Institute for Health Research’s (NIHR) INVOLVE recently identified five key principles for co-production [23]. These provide a useful critical framework for reflection.

### Sharing of power

Young people took on considerable responsibilities throughout the project. They generated the original idea, interviewed CRC and accepted them as partners, set the tenor and overall message of the film, decided on the primary audience and generated original audio and visual material based on their own experiences and ideas. Editing decisions were based on the views and wishes of the young people. The rough cut review meeting provided opportunities to input into the final cut.

### Include perspectives and skills of all those who can make a contribution

Working with young people from established participation groups risks excluding the most seldom heard voices, whose views are often absent from debate. In this project only three young people were members of the IMPACT-ME steering group, others were invited from the wider study sample. The group was ethnically diverse although only two of the seven were male. The film reflects the experiences of this small group of motivated young people. We cannot claim that it represents the experiences of all young people.

The four-day commitment may also act to exclude some young people. Shorter sessions held over several weeks, perhaps in the evenings, may be more manageable for some. However, the four-day block enabled momentum to build, relationships, trust and team spirit to grow and ideas to flourish. Balancing the ideal with the convenient is a challenge.

On two occasions, when young people were unable to attend a particular session, we were able to arrange a bespoke session outside the four-day workshop. This flexibility ensured we were able to be inclusive. However, flexibility is expensive and time consuming. Contingency funds and a generous timeframe are essential.

### Respect and value the knowledge of all - everyone is of equal importance

We were aware of the sensitivity of the topic under discussion and that group discussion may be difficult for some young people. We offered young people a range of options, both audio and visual through which they could contribute. A designated quiet space enabled young people to record conversations in pairs away from the main hub of activity. Young people with a specific interest or skill were able to focus on or develop their interest if they so wished. For example, one young person, a skilled artist with a passion for animation, worked closely with LH. Another young man wrote the Facing Shadows guitar track.

### Reciprocity - everybody benefits from working together

This creative, informal, collaboration offered young people an opportunity to focus on their creativity and strengths. They were brimming with ideas and insights which they were eager to communicate. TM reflected, *‘we’ve seen, and young people have told us, how much confidence they gain from working together towards shared goals’.* The young people were proud of having created a resource for other young people, as well as having developed practical skills. The researchers gained new insights into depression in young people and their experiences of therapy. For example, the visual metaphors that emerged in the creative workshops were a powerful graphic addition and stimulated insightful conversations about depression. Although young people held strikingly different views of therapy (some very positive, others quite dissatisfied), they were united in the view that the film should convey the message that it is always better to talk and seek help.

The young people acknowledged the learning opportunities that the workshop had offered, ‘*it was interesting because they brought in lots of equipment like lighting and cameras that I hadn’t used before, so it was fascinating to learn how to use something new*’.

The role of the adults was to facilitate, guide, teach, support and respect the young people’s skills and experiences in order to maximise their involvement.

### Build and maintain relationships - relationships is key to power sharing

Relationship building is the bedrock of the project. Trusting relationships, built on mutual respect are at the heart of power sharing, decision-making and learning.

The project brought together three distinct groups, each with an established identity and distinct sets of skills, experiences and expertise: the research team had worked together on the IMPACT-ME study and brought insider knowledge and insights from the study, research skills, mental health knowledge and a commitment to enabling young people to achieve their aims; the creative team combined film-making, group work, art and research skills and experience employing these with young people in creative research settings; the young people brought their personal experiences of moderate/severe clinical depression and therapy and a desire to use their insights to offer help and hope to other young people.

As confidence and trust developed between the groups, as common ground was established and aims were agreed, a new, whole-group identity emerged - that of film-makers. This new identity recognised the value of sharing personal assets and skills, mutual respect and co-production.

The young people valued the relationships, ‘*everyone’s been really open, I’ve never felt so comfortable with, like, a group of people before’*. They felt the adult involvement had been genuine, ‘*the most important thing was that the adults were really interested in what the young people had to say, which is a situation you don’t find yourself in very often’*.

## Limitations

Although young people were fully involved at most levels of the project, we did not offer the opportunity to co-author publications. At the outset, we sought funding for 12 months, sufficient only to cover the coproduction. In retrospect, an extra six months funding would have enabled us to offer a broader range of involvement opportunities, including co-writing for publication.

The workshops generated a great deal more material than could be accommodated in the film. We could have made better use of this, for example by producing materials and learning resources to assist teachers or trainers to make the most of the film in schools or clinical training institutes.

Increased funding for longer, would also have enabled us to monitor the film’s impact and feed this back to young people.

Ideally, we should have arranged, and funded, an independent evaluation of the project to maximise the learning potential to inform future projects.

## Budget

Funding of £2000 was secured from a Beacon Bursary grant from University College London which, together with £10,000 from the Monument Trust, met the CRC fees, young people’s expenses, refreshments and film launch event venue hire. Workshops were held, at no cost, at the Anna Freud National Centre for Children and Families.

## In conclusion

The workshop approach, rooted in creative PR, transformed our project from dissemination of research findings to a stand-alone exploration of depression and therapy as experienced by a group of young adults.

Such approaches require thorough planning, commitment, people, resource and time [24], which should not be underestimated. However, they have the transformative capacity to empower vulnerable young people to influence the services, institutions and decisions which affect their lives.
